# Biomechanical evaluation of fixation of the coracoclavicular stand-alone cow-hitch suture reconstruction in comparison to two established techniques for highly unstable distal clavicle fractures (Neer type V)

**DOI:** 10.1016/j.jseint.2023.11.022

**Published:** 2024-01-02

**Authors:** Paul Borbas, Alexander Paszicsnyek, Simon Hofstede, Lukas Ernstbrunner, Karl Wieser

**Affiliations:** a Department of Orthopaedics, Balgrist University Hospital, Zurich, Switzerland; b Institute of Biomechanics, ETH Zurich, Zurich, Switzerland

**Keywords:** Distal clavicle fracture, Coracoclavicular ligaments, Coracoclavicular instability, Cow-hitch, Suture reconstruction, Biomechanical properties

## Abstract

**Background:**

Treatment of displaced distal clavicle fractures with bony avulsion of the coracoclavicular (CC) ligaments often warrants surgical fixation, yet a gold standard surgical technique is to be defined. The purpose of this study was to compare the biomechanical fixation strength of a new fixation technique, the CC stand-alone cow-hitch suture reconstruction, and to compare this technique with a clavicle hook plate and a lateral locking plate with CC suture reconstruction.

**Methods:**

Simulated Neer type V distal clavicle fractures of the clavicle were created in 18 cadaveric shoulders, which were matched by age and gender in 3 groups: (1) clavicle hook plate (group HP), (2) lateral locking plate fixation with CC suture reconstruction (group LPCC), and (3) CC stand-alone suture reconstruction using the cow-hitch technique (group CH). After preconditioning with 25 N for 10 cycles, the specimens were cycled in the coronal plane for 500 cycles from 10N to 70N. Displacement and ultimate load to failure were documented and analyzed with the data acquisition system.

**Results:**

There was a significant difference in the fracture displacement during cyclic loading between the LPCC group and the HP group (0.6 vs. 1.7 mm; *P* = .02) and between the CH and HP groups (0.5 vs. 1.7 mm; *P* = .004). Fracture displacement was not different between the LPCC and the CH groups (*P* = .544). The CH group and the LPCC group showed a significantly higher stiffness compared to the HP group (*P* < .001 and *P* = .003, respectively). The CH group showed a significantly higher ultimate load to failure compared with the HP group (429 vs. 172 N; *P* = .005) and showed a tendency toward higher ultimate load to failure when compared with the LPCC group (429 vs. 258 N; *P* = .071).

**Conclusion:**

The CC stand-alone cow-hitch suture reconstruction and the locking plate with CC reconstruction showed higher fixation strength compared with the hook plate for simulated Neer type V distal clavicle fractures. There was a tendency of higher ultimate load to failure with the cow-hitch technique compared with the lateral locking plate with CC suture reconstruction, and given the potential advantages of less soft tissue stripping, metal-free fixation, low costs, and simple surgical technique, clinical application of the all-suture CC reconstruction using the cow-hitch for Neer type V distal clavicle fractures appears warranted.

The treatment of displaced distal clavicle fractures remains challenging, especially for Neer type V fractures.^21^ Such fractures are highly unstable and are associated with a high nonunion rate when nonoperative management was chosen.^3^

Surgical fixation is usually preferred in these fractures due to the given instability and high malunion and nonunion rates but a single gold standard technique is yet to be defined.^2^^,^^8^^,^^15^^,^^21^^,^^22^^,^^25^ Clavicle hook plate fixation is one of the most commonly performed surgical procedures in these fractures due to its easy applicability and high union rates.^20^^,^^24^^,^^30^ However, drawbacks of this technique include the necessity of plate removal as retaining the implant may result in acromial erosion and fracture or rotator cuff tendinopathy.^13^^,^^19^^,^^24^^,^^30^^,^^31^ An alternative to that is the application of a lateral locking plate with or without coracoclavicular (CC) stabilization, which also achieved relatively high union rates but requires relatively extensive soft tissue stripping, is expensive and may not achieve sufficient fixation in the lateral fragment.^33^ A new, simple, and relatively cheap concept to stabilize distal clavicle fractures is an all-suture CC reconstruction technique using cow-hitch knots.^6^ First clinical and biomechanical results of this all-suture suture technique to fix distal clavicle fractures were promising,^6^^,^^17^ but its performance for complex, highly unstable distal clavicle fractures (Neer type V) is unknown. It was therefore the aim of this biomechanical study to compare the fixation strength of the CC stand-alone suture reconstruction using the cow-hitch technique with hook plate fixation and with lateral locking plate fixation with additional CC stabilization for Neer type V distal clavicle fractures. In a non-inferiority study design, we hypothesized that the cow-hitch fixation technique will show equal fixation strength compared with the hook plate and lateral locking plate fixation techniques.

## Methods

For this biomechanical study, 18 fresh frozen human cadaveric shoulders (12 female, 6 male) were obtained from Science Care (Phoenix, AZ, USA), with an average age of 70.6 ± 3.4 years. Computertomography scans were used to ensure the integrity of the clavicle specimens and, in addition, a measurement of bone density (Hounsfield units) was performed in a 5 × 5 mm area above the conoid tubercle of the clavicula. The 18 shoulders were matched in the three groups in terms of age, bone quality, and sex.

### Specimen preparation

The cadaveric shoulders were thawed at room temperature 24 hours prior to dissection. The skin and the subcutaneous tissue were removed and any muscles were detached from the scapula and clavicle, with preservation of the CC ligaments and the joint capsule of the acromioclavicluar (AC) joint. The glenohumeral joint was then disarticulated. During dissection, the cadavers were kept regularly moist with phosphate-buffered saline solution to avoid desiccation before, during, and after the testing process, as well as surgical dissection.

**Figure 1:**
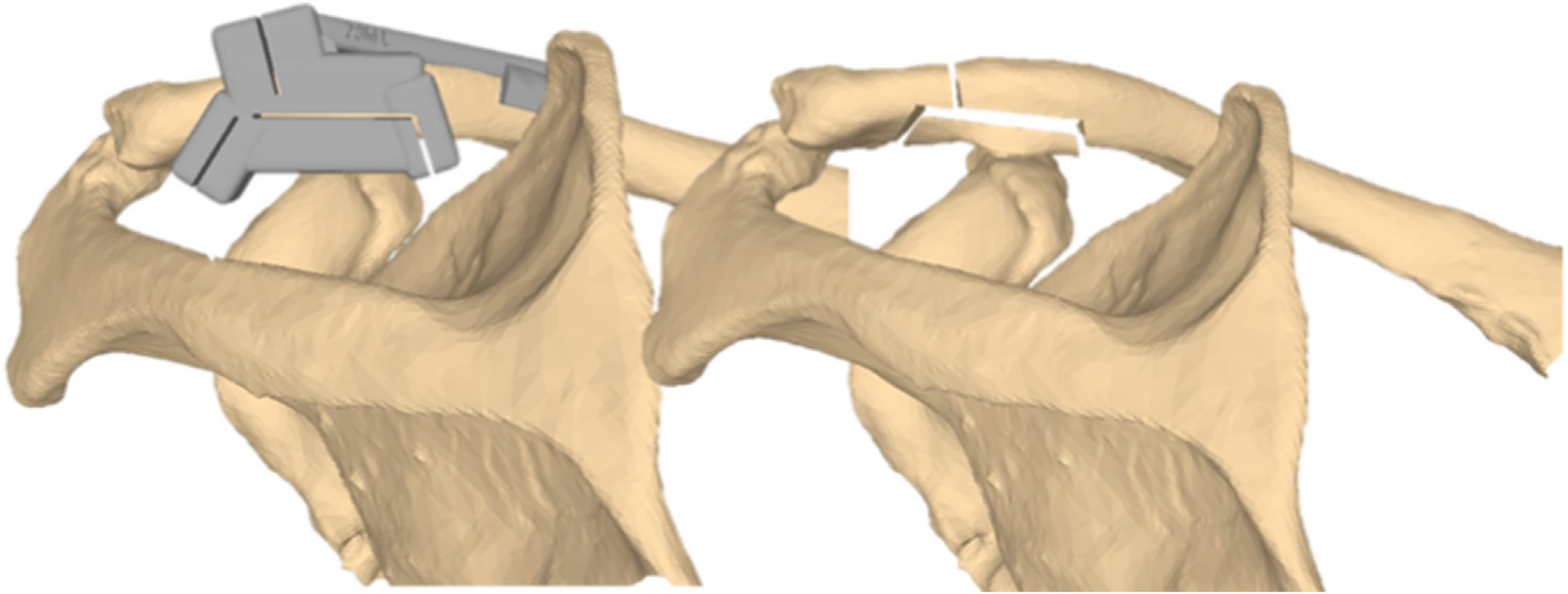
Placement of the preplanned cutting block on the 3D model of one included clavicle and simulated fracture configuration after cutting according to Neer type V fractures.^21^

All surgeries were conducted by a fellowship-trained shoulder surgeon (P.B.). For the setting of the Neer type V fractures, standardized 3D-printed cutting guides were planned for each individual specimen using segmented computertomography scans in a 3D planning program (CASPA, Balgrist CARD AG, Switzerland). These allowed reproducible Neer type V fracture placement in 4 cutting planes (Fig. 1). The horizontal fracture was set at one-third of the bone height of the clavicle in the transverse plane. The superior osteotomy was set at one-fourth of the horizontal cut at 20° to the plane to allow fixation from proximal. The medial and lateral osteotomies were set with respect to the extent of the CC ligaments, as well as the anatomical landmarks (conoid tubercle). 3D printing of the cutting guides was performed by Medacta International SA (Castel San Pietro, Switzerland). The fractures were set using an oscillating saw. Before the final treatment of the fracture, the fracture segments were temporarily stabilized with pointed reduction forceps and then finally treated with the respective technique according to the group assignment. To verify the anatomically correct reduction and correct fixation, radiographs were taken in the anterior-posterior and lateral planes using a mobile image intensifier.

### Fracture fixation

#### Group 1: hook plate fixation (group HP)

3.5 mm LCP Clavicle Hook Plates (DePuy Synthes (Johnson & Johnson), New Brunswick, USA) were used and osteosynthesis was performed according to the manufacturer’s surgical technique brochure. The fracture was anatomically reduced with pointed reduction clamps followed by temporary fixation with K-wires. The plate was positioned over the clavicle with the distal end ending up just proximal to the AC joint and the hook being placed posterior to the clavicle and under the acromion. One 3.5 mm cortical screw and one 3.5 locking screw were inserted into the plate medial to the fracture and two 3.5 locking screws were inserted into the plate lateral to the fracture.

#### Group 2: lateral locking plate fixation with CC suture reconstruction (group LPCC)

Distal clavicle fracture plates (Arthrex, Naples, FL, USA) were used with CC ligament stabilization using TightRope and Dog Bone Buttons, according to the user manual. The fracture was anatomically reduced with pointed reduction clamps followed by temporary K-wire fixation. The plate was then positioned on the clavicle and one 3.5 mm cortical and three locking screws were inserted medially and five 2.7 mm locking screws laterally. Finally, CC fixation with a TightRope was performed after drilling through the clavicle and the base of the coracoid process just anterior to the CC ligaments with a 3.7 mm drill bit. The TightRope loop, which was secured by a Dog Bone button under the coracoid and another vbutton that fits into the plate superiorly, was hand-tightened.

#### Group 3: stand-alone suture reconstruction using the cow-hitch technique (group CH)

The fracture was anatomically reduced with pointed reduction forceps followed by temporary fixation of Kirschner wires. A bicortical hole was drilled between the attachment of the two CC ligaments through the coracoid process and a Dog Bone button loaded with two 2 FiberWire #5 (Arthrex, Naples, FL, USA) was placed at the coracoid bottom and the sutures were passed through the coracoid drill hole. Finally, two clavicular drill holes were created with a 2 mm drill bit. These were located at a distance from each other approximately 20 mm medial to the superior fracture. One FiberWire suture was passed through each of the medial and lateral drill holes in a loop constellation. After implementation, there were thus two loops above the clavicle and four suture ends below for the implementation of two cow-hitch knots, which were placed midway between the underside of the clavicle and the coracoid. The anatomical reduction of the fracture was rechecked and the cow-hitch knots were finally tightened and secured with three alternating half-hitch knots.

### Experimental testing

**Figure 2:**
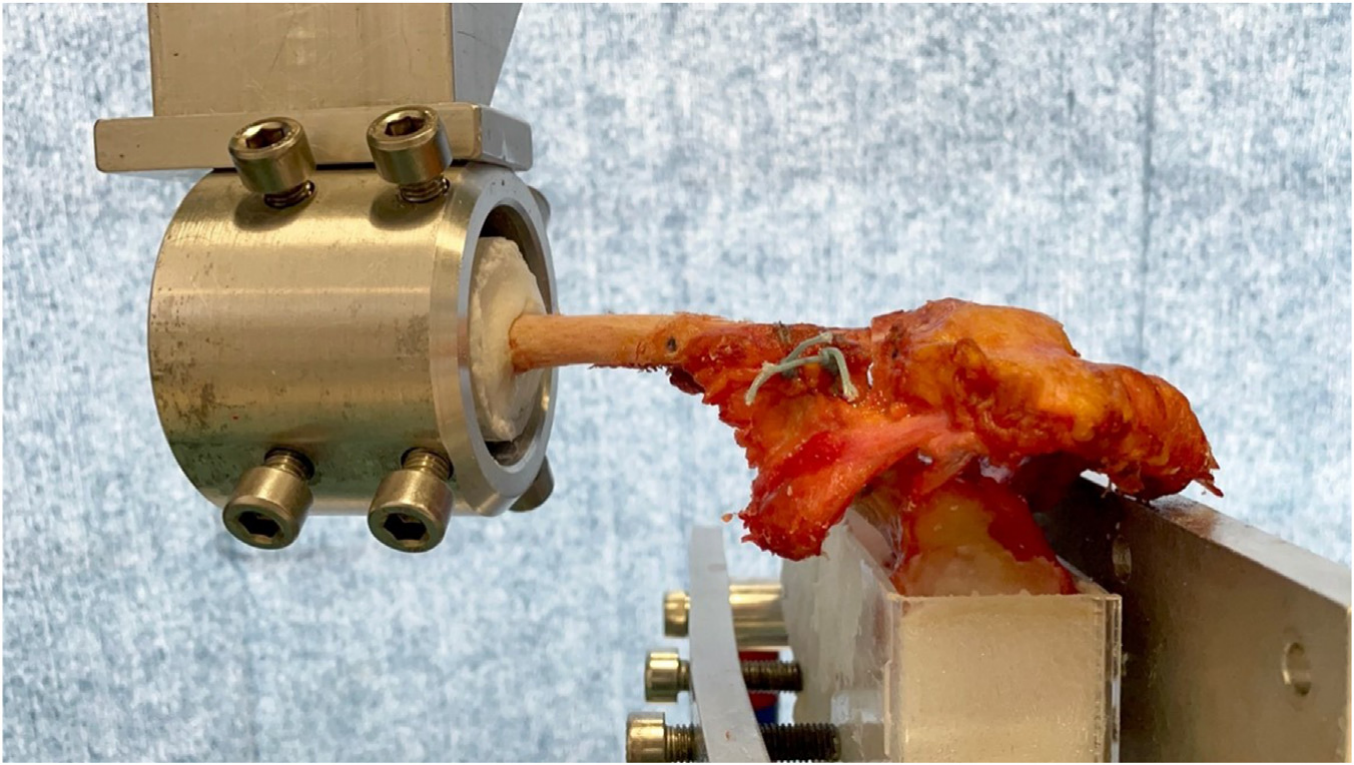
Experimental testing setup with the clavicle mounted to the machine using a polyvinyl chloride tube and anchored using bone cement.

A servo-hydraulic machine for materials testing (Zwick-Roell, Ulm, Germany) was used for the testing; the respective data were recorded in dedicated software (testXpert III, Zwick-Roell GmbH & Co, Ulm, Germany) and analyzed for the applied load (force (N) and displacement (mm)). A standardized protocol, similar to previous biomechanical studies^1^^,^^33^ on distal clavicle fractures, was used for the study procedure. For adequate fixation during testing, the proximal clavicle was centered in a polyvinyl chloride tube and then anchored using bone cement. The scapula was also fixed caudally in a box with bone cement. A horizontal plane starting from the center of the glenoid cavity served as a landmark for fixation. For proper performance, care was taken to position the clavicle anatomically with respect to the scapula and to create anatomical tension in the AC joint (Fig. 2). The cadavers were preconditioned from 10 N to 25 N for 10 cycles and then tested from 10 N to 70 N at 1 Hz frequency in the coronal plane for 500 cycles. After the cyclic testing, another protocol was applied to determine the force and displacement at ultimate load-to-failure with a speed of 1 mm/s. Failure was defined as a force drop of 50% on the curve.

To obtain another control parameter, a MicroScribe Digitizer M (Immersion, San Jose, CA, USA) was used after the determination of two fixed and marked points on the clavicle lateral and medial to the fracture. Using these points, the displacement relative to each other and their localization (at 0 N before preconditioning, at 0 N and 70 N before and after cyclic testing) is accurately represented in a coordinate system (virtual, 3D). The MicroScribe Digitizer M represents a precise additional component for testing, which illustrates the values in three-dimensional space with a statistical error of 0.05 mm.

### Statistical analysis

IBM SPSS Statistics 26 software (IBM Corporation, Armonk, NY, USA) was used for statistical analysis of the data. A power analysis was carried out deeming 6 specimens per group as sufficient for a significant difference if found. Normal distribution was determined with the Shapiro-Wilk test. Data were presented using mean and standard deviation values. Comparison of all 3 groups was performed using analysis of variance (ANOVA; parametric data) or the Kruskal-Wallis test (nonparametric data). Bonferroni correction (ANOVA) and Dunn-Bonferroni correction (Kruskal-Wallis) were used and significance was set at *P* < .05, with all *P* values two-sided.

## Results

Due to an incorrect fracture reduction, one specimen from the CH group had to be excluded from the statistical analysis.

No significant difference was found between the 3 groups regarding the bone quality of the distal clavicle using Hounsfield units (HP group: 109.7 HU; LPCC group: 114.8 HU; CH group: 116.1 HU; *P* = .636).

During cyclic testing, group HP showed statistically significant higher fragment displacement compared to group LPCC (0.6 vs. 1.7 mm; *P* = .02) and to group HP (0.5 vs. 1.7 mm; *P* = .004). No significant displacement difference could be determined between group LPCC and group CH (0.6 vs. 0.5 mm; *P* = .544). In terms of stiffness (N/mm), there was also a significant difference between the groups (*P* < .001). Here again, group LPCC (65.9 vs. 16.8 N/mm; *P* = .003) and group CH (83.2 vs. 16.8 N/mm; *P* < .001) each showed significantly higher stiffness than group HP. No significant difference was shown between group LPCC and group CH (65.9 vs. 83.2 N; *P* = .357).

At ultimate-load-to-failure testing, a statistically significant difference was found between the 3 groups (*P* = .006). Group CH showed a significantly higher ultimate breaking load compared to group HP (428.7 vs. 171.5 N; *P* = .005). Comparison of group LPCC with group CH (258.2 vs. 428.7 N; *P* = .071) and of group LPCC with group HP was not statistically significant (258.2 vs. 171.5 N; *P* = .661).

The clavicle displacement at ultimate failure showed no significant statistical difference across all groups (*P* = .063). The greater displacement in group HP compared to group LPCC was not statistically significant (17.7 vs. 8.1 mm; *P* = .067), as did the differences between group LPCC with group CH (8.1 vs. 11.4 mm; *P* = 1), and between group CH and group HP (11.4 vs. 17.7 mm; *P* = .344).

Detailed results are listed in Table 1.

**Table I:** Data for the included groups during and after cyclic testing.

	Group 1: HP	Group 2: LPCC	Group 3: CH	P*ƒ*
Fracture displacement after cyclic testing (mm)	1.7 ± 0.3	0.6 ± 0.2	0.5 ± 0.2	0.012
Stiffness (N/mm)	16.8 ± 2.6	65.9 ± 11.6	83.2 ± 26.5	<0.001
Ultimate LTF ⊗ (N)	171.5 ± 67.7	258.2 ± 76	428.7 ± 176	0.006
Distance to LTF (mm)	17.7 ± 9.1	8.1 ± 3.1	11.4 ± 5.8	0.063

*HP*, clavicle hook plate; *LPCC*, lateral locking plate fixation with CC suture reconstruction; *CH*, stand-alone cow-hitch suture; *ANOVA*, analysis of variance; *LTF*, load to failure.

All data are presented using mean ± standard deviation.

ƒ, For comparison of the groups ANOVA was used for parametric data.

⊗, defined as force drop of 50% on the curve.

With regard to the failure mechanism, differences were observed between the groups. Failure at the level of the medial screw of the plate was most frequently seen for group HP (n = 5). For group CH, failure at the medial drill hole was documented as the most common mechanism (n = 4). Group LPCC had a migration of the button into the coracoid process as the most common failure mechanism (n = 4).

Details about failure mechanisms can be seen in Table 2.

**Table II:** Detailed list of failure mechanisms for all included groups.

Group 1: HP∗	Group 2: LPCC	Group 3: CH
Fracture at level of medial screw (n = 5)	Migration of the coracoid button (n = 4) Fracture at medial screw (n = 2)	Fracture at medial clavicular drill hole (n = 4) Fracture at both clavicle drill holes simultaneously (n = 1) Fracture at cement-bone transition (n = 1)

*HP*, clavicle hook plate; *LPCC*, lateral locking plate fixation with CC suture reconstruction; *CH*, stand-alone cow-hitch suture.

∗ Exclusion of 1 specimen due to insufficient fracture reposition.

## Discussion

The main finding of this study is that the CC stand-alone cow-hitch suture reconstruction produces equivalent or superior fixation than the lateral locking plate with CC reconstruction, and both are biomechanically stronger than a hook plate for Neer type V distal clavicle fractures. Therefore this treatment strategy may yield a new sufficient approach for fixation.

The treatment of unstable distal clavicle fractures is challenging. Several treatment options have been described in previous studies, but none of the techniques has emerged as a superior option.^5^^,^^7^^,^^11^^,^^14^^,^^23^^,^^32^ Therefore, the aim of this study was to investigate the biomechanical stability of three treatment options for Neer type V fractures. Fracture fixation with a hook plate or a lateral locking plate with CC reconstruction are commonly used methods with generally reliable union rates and good clinical results.^10^^,^^20^ We compared these with a simple CC suture reconstruction technique, the stand-alone cow-hitch technique, which has been introduced for AC joint stabilizations and distal clavicle fractures and was evaluated regarding biomechanical stability in Neer type II fractures with good results.^6^^,^^16^^,^^17^ Previous biomechanical studies focused mainly on type II fractures.^16^^,^^33^ A recent study by Laux et al^16^ compared the cow-hitch suture reconstruction technique with the well-established suture tape double-button fixation for Neer type IIB fractures, with the cow-hitch suture reconstruction showing promising and noninferior results. Therefore, this fairly novel, simple, and cost-effective technique^6^ can be considered as a treatment option for Neer type V fractures as well. However, Neer type V fractures are particularly challenging since they are multifragmentary with a bony avulsion of the CC ligaments. The proposed cow-hitch technique allows besides the CC fixation for an additional interfragmentary compression between the CC avulsion fragment and the clavicle shaft. We further assume the interfragmentary closed loop configuration reduces the stress load on the suture button configuration by transferring the stress to the intact CC ligaments.

The clavicular hook plate showed the lowest ultimate load to failure in this study, which was significantly lower compared to the stand-alone cow-hitch technique and the lateral clavicle plate with CC stabilization. This could be related to the lack of CC stabilization, which was performed with the other two techniques. Furuhata et al found significantly higher rates of CC insufficiency in Neer type V fractures compared to Neer IIa fractures after locking plate fixation without CC reconstruction.^9^ These findings also support the importance of CC stabilization in Neer type V fractures. In previous biomechanical studies for Neer type IIB fractures, it was shown that fixation at the coracoid process is an important component for the construct stability. Yagnik et al^33^ showed in their study that a CC stabilization, with or without allograft supply, has up to 75% greater biomechanical stability than a lateral clavicle plate alone in Neer type IIB fractures. Alaee et al^1^ also investigated different treatment techniques for unstable clavicle fractures, but all used a lateral clavicle plate with different fixation to the coracoid process. No difference was found between the techniques, but the results showed a higher stiffness compared to the native CC ligaments, and a generally higher stability when using CC stabilization compared to a plate alone.

Plating of unstable clavicle fractures generally presents with a high complication potential. Revision surgery is one of the most relevant complications, especially with the hook plate since the plate has to be removed in 63-100% of cases.^23^^,^^29^ All of these factors may affect the subjective and objective patient-specific outcome.^23^ There are also implant-specific complications for the lateral clavicle plate with CC stabilization. The migration of the button into the coracoid process and the resulting coracoid fracture leads to inferior outcome and satisfaction after the surgical treatment.^34^ Furthermore, 29-42% of cases require revision surgery due to irritation and material failure^23^^,^^27^ and for the fixation of the plate, the lateral fragment of the fracture must be large enough to provide sufficient screw placement for adequate fixation.^12^^,^^28^

The significantly higher stiffness values for the stand-alone cow-hitch technique compared to the clavicular hook plate can probably be attributed to the configuration of the knots and threads as well as the leaflet-like configured Dog Bone Button.^18^ Through the special knot technique and the used button, the applied force is distributed more evenly onto the coracoid. This might justify the better result for the stand-alone cow-hitch group and the lateral clavicle plate with CC stabilization group. No significant differences in terms of stiffness were to be found between the stand-alone cow-hitch and the lateral clavicle plate with CC reconstruction group.

In the hook plate group, fracture at the medial screw was the most common failure mechanism. A similar failure mechanism medial to the plate or at the medial screw was described in a previous biomechanical study.^4^ In the lateral clavicle plate with CC reconstruction, migration of the button into the coracoid process was shown to be the most common failure mechanism. Due to its design, which is very flat and narrow, the traction acts on a comparably small area. Superior button migration into the coracoid process has been previously described as a complication after AC joint stabilization using a TightRope technique.^26^ This may be avoided by using a flat button with a bigger surface, as in the stand-alone cow hitch technique, so that the traction could be more evenly distributed. In our biomechanical study, the most common failure mechanism in the stand-alone cow hitch group was fracturing at the medial drill hole of the clavicles.

The present study has various limitations. First of all, since this is an experimental study on human shoulder cadavers, fixation strength of the proposed techniques might differ in actual patients. Although 6 specimens per group (18 shoulders in total) is a reasonable number and significant differences were observed, the groups might still be underpowered, which may also explain why the higher ultimate load to failure in the CH group compared with the LPCC group showed only a statistical trend but no actual significant difference. However, as this study was designed as a non-inferiority study the sample size seems to be sufficient. Simulating fractures with 3D-printed guides is an elaborate approach to achieve comparable fracture patterns within the examined shoulder specimens. Nevertheless, standardized fracture simulation does not resemble in-vivo fracture patterns, which might have a relevant, but with our setup not reproducible, effect on fixation strength. Lastly, we only tested vertical but no horizontal displacement. Although horizontal instability is known to be more of an issue in acromioclavicular joint dislocations, the primary fixation of distal clavicle fractures with bony avulsion of the CC ligaments has also to resist anteroposterior directed forces, which may warrant further biomechanical testing in future studies.

## Conclusion

The CC stand-alone cow-hitch suture reconstruction provides sufficient fixation strength for Neer Type V fracture. Given the potential advantages of less soft tissue stripping, metal-free fixation, low costs, and simple surgical technique, clinical application of the all-suture CC reconstruction using the cow-hitch for Neer type V distal clavicle fractures appears as a possible approach. Further clinical studies for the validation of this outcome are needed.

## Disclaimers:

Funding: No funding was disclosed by the authors.

Conflicts of interest: The authors, their immediate families, and any research foundation with which they are affiliated have not received any financial payments or other benefits from any commercial entity related to the subject of this article.
